# Association of eating out of home and type 2 diabetes mellitus in Chinese urban workers: A nationwide study

**DOI:** 10.1002/cdt3.136

**Published:** 2024-06-13

**Authors:** Fangyan Chen, Sitong Wan, Jinjuan Hao, Ke Sun, Annan Liu, Ling Zhu, Shuyan Wang, Jingjing He, Ping Zeng

**Affiliations:** ^1^ The Key Laboratory of Geriatrics, Beijing Institute of Geriatrics, Institute of Geriatric Medicine, Chinese Academy of Medical Sciences Beijing Hospital/National Center of Gerontology of National Health Commission Beijing China; ^2^ Department of Nutrition and Health China Agricultural University Beijing China; ^3^ Hospital Administration Office, Beijing Hospital, National Center of Gerontology, Institute of Geriatrics Medicine Chinese Academy of Medical Sciences Beijing China; ^4^ Department of Neurology, Xuanwu Hospital Capital Medical University Beijing China

**Keywords:** dose–response relationship, eating out, type 2 diabetes, urban workers

## Abstract

**Background:**

The prevalence of type 2 diabetes mellitus (T2DM) has been rapidly growing in Chinese populations in recent decades, and the shift in eating habits is a key contributing factor to this increase. Eating out of home (EOH) is one of the major shifts in eating habits during this period. However, the influence of EOH on the incidence of T2DM among Chinese urban workers is unknown.

**Methods:**

The cross‐sectional study involved an analysis of 13,904 urban workers recruited from 11 health examination centers in the major cities of China to explore the relationship between EOH and T2DM between 2013 September and 2016 March.

**Results:**

Average weekly EOH frequency ≥10 times was positively associated with increased incidence of T2DM in the sampled population (OR: 1.31 [1.11–1.54], *p* < 0.01), most notably in participants ≤45 years old (OR: 1.41[1.11–1.80], *p* < 0.01]) and in males (OR:1.26 [1.06–1.51], *p* < 0.01). An EOH frequency of 5 times/week appears as a threshold for a significant increase in the odds of T2DM. Weekly EOH frequency ≥5 times was associated with increased odds of T2DM in a dose–response manner in the total population and almost all subgroups (*p*_overall association_ < 0.05 and *p*_nonlinearity_ ≤ 0.05).

**Conclusion:**

This study showed that a frequency of EOH (≥5 times/week) was associated with a frequency‐dependent increase in the odds of T2DM urban workers in China. More nutrition promotion is needed to improve the eating behavior of Chinese urban workers to reduce T2DM risk.

## Highlights

- High frequency of eating out of home (EOH) was associated with increased odds of type 2 diabetes mellitus (T2DM) in Chinese urban workers.
- The positive association between EOH and T2DM was independent of sex, and the association was strongest in individuals ≤45 years of age.
- EOH frequency of about 5 times/week seemed to be a threshold for the increased odds of T2DM.

## Graphical abstract

Dose–response associations between weekly frequency of eating out of home (EOH) and type 2 diabetes mellitus (T2DM) in (A) the total population, (B) male population, (C) female population, (D) population > 45 years old, and (E) population ≤45 years old. The fully adjusted model included age, gender (except for male and female population), BMI, smoking status, drinking status, physical activity, hypertension, dyslipidemia, and family history of T2DM.

## 1 INTRODUCTION

Type 2 diabetes mellitus (T2DM) is a globally multifactorial disease, affecting 537 million individuals in 2022.^1^ The prevalence of T2DM has been rapidly growing in Chinese populations in recent decades, especially in adolescents and young adults.^2^, ^3^ The major risk factors for T2DM include a higher body mass index (BMI), a suboptimal diet (e.g., low fruit intake and high red meat or processed meat intake), smoking, and low physical activity.^3^ Among these risk factors, suboptimal diet has been shown to be the key contributing factor to T2DM‐related death and disability in Chinese populations.^4^ Many of these dietary changes have been attributed to the shift from traditional eating habits in China toward more Westernized diets.^5^

Eating out of home (EOH) has been one of the major changes in lifestyles in recent decades. EOH is associated with the consumption of a variety of nontraditional food types (e.g., restaurant food, delivery food, and take‐out food) and is becoming more and more popular in many different countries.^6^ Although EOH provides more convenience and a greater range of food choices, it has been regarded as an important risk factor for metabolic diseases because of its high caloric and sodium content but suboptimal levels of micronutrients.^7^, ^8^, ^9^ Findings from observational studies have shown that increased frequency of EOH is associated with weight gain in adults^9^, ^10^ and increased incidence of childhood obesity.^10^, ^11^, ^12^ Higher frequencies of EOH have also been associated with increased odds of high blood pressure and blood lipid abnormalities in the Korean population.^13^

As a lifestyle‐related disease, T2DM has also been reported to be associated with EOH. In the United States, evidence from the Nurses' Health Study and Health Professionals Follow‐up Study showed that higher EOH frequency was associated with elevated T2DM risk.^14^ Findings from Chinese rural workers in one province suggested a positive association between EOH and T2DM in males but not in females.^6^ A national health and nutrition survey from 2004 to 2011 showed that urban workers have a higher frequency of EOH than rural workers in China.^15^ It means EOH may generate more impact on the health status of urban workers, whose lifestyle and diet patterns have been significantly shifted in the past few decades. However, few studies have explored the influence of EOH on the occurrence of T2DM in Chinese urban workers. In this study, we have used data from multiple centers across the nation to explore the relationship between EOH and T2DM among 13,904 urban workers who received medical examinations. Our goal is to raise awareness of potential health risks that may exist.

## 2 METHODS

### 2.1 Study population

The sample size was calculated using the following formula: $\mu^{2}p(1-p)/d^{2}$, in which the confidence level is taken as 95% (two‐sided), and the corresponding *u* = 1.96. The probability *p* is taken as China's national monitoring diabetes prevalence rate of 9.7% in 2010,^16^ the relative error *r* = 20%, and *d* = 20%×9.7% = 0.0194. Based on the values of the parameters mentioned above, it is calculated that the sample size of each stratum on average is about 895 persons. According to the number of 11 strata and taking into account the 20% nonresponse rate, the total sample size was calculated to be about 12,000 persons.

This study included medical examination data from 17,760 Chinese urban workers collected between September 2013 and March 2016 who were enrolled in 11 health centers in major cities, including Beijing, Chongqing, Hangzhou (Zhejiang Province), Jinan (Shandong Province), Changsha (Hunan Province), Fuzhou (Fujian Province), Wuhan (Hubei Province), Hefei (Anhui Province), and Nanjing (Jiangsu Province). After excluding participants with current or a history of severe diseases (e.g., cancer, physical disabilities, coronary heart disease, stroke, dementia, severe psychological disorders, tuberculosis, or other communicable diseases) or for whom critical data were missing, a total of 13,904 mentally active participants were included in the final study population.

The study protocol was reviewed and approved by the ethics committee of Beijing Hospital (BJYYEC‐KY104‐01) and implemented according to the principles of the Declaration of Helsinki. All participants gave their informed consent before data collection.

### 2.2 Blood sample collection and analysis

In the early morning, 3‐mL venous blood was drawn from the participants who had been fasting for at least 8 h and placed into the EDTA anticoagulant tube for detection of fasting blood glucose (FBG), glycated hemoglobin (HbA1c) and blood lipids (triglycerides [TG], total cholesterol [TC], high‐density lipoprotein cholesterol [HDL‐C], and low‐density lipoprotein cholesterol [LDL‐C]), measuring enzymatically following standard methods with a modular P800 model autoanalyzer (Roche) with reagents (Roche Diagnostic GmbH). Then, participants drank 250 mL of water containing 75 g of glucose, and 3‐mL venous blood was drawn after 2 h for detection of 2‐h oral glucose tolerance test blood glucose (OGTT 2 h).^17^

### 2.3 Definition of EOH and T2DM

EOH was defined as having meals outside the home, including restaurant food, canteen food, delivery food, take‐out food, and food services.^6^ The participants were surveyed by trained physicians to report their average EOH frequency in a 1‐week period. EOH frequency was then classified into <5, 5–9, and ≥10 times per week in the analysis, considering generally 5 days as working days, so that EOH = 5 indicated having five meals EOH per week.

T2DM was diagnosed as FBG ≥ 7.0 mmol/L or 2‐h OGTT 2 h blood glucose ≥ 11.1 mmol/L.^18^

### 2.4 Measurement of covariates

Questionnaires containing general demographic information such as age, gender, health status, individual and family history of diseases, medications, and lifestyle factors were administered to participants. Smoking was defined as smoking ≥ 1 cigarette/day for 6 months or more. Alcohol drinking was defined as drinking ≥ 1 time/week for 6 months or more. Any participant who had ever (currently or previously) fit the study's definition of a smoker or drinker was defined as a smoker or drinker for the purposes of this study. Based on the International Physical Activity Questionnaire Short Form, physical activity level was measured as metabolic equivalents‐min/week and then classified into low, moderate, and high according to the tertiles.^19^ Anthropometric measurements including height, weight, and waist circumference were obtained by trained physicians. BMI (kg/m^2^) was calculated as weight in kilograms divided by the square of height in meters. Using a sphygmomanometer after a 5‐min rest, blood pressure was measured two times for each participant with a minimum 30‐second break between measurements, and the average of the two readings was calculated. Hypertension was defined as systolic blood pressure (SBP) ≥ 140 mmHg or diastolic blood pressure (DBP) ≥ 90 mmHg.^20^ Dyslipidemia was defined as TG ≥ 2.3, TC ≥ 6.2, LDL‐C ≥ 4.1, or HDL‐C < 1.0 mmol/L.^21^

### 2.5 Statistical analysis

Data were reported as means ± standard deviations (SD) for continuous variables and counts (percentages) for categorical variables. Independent samples *t* test or Mann–Whitney *U* test was used to compare the continuous variables, and the χ^2^ test was used for the comparison of categorical variables.

The association between EOH and T2DM was analyzed in the total sampled population, men, women, people younger than 45 years old, and people older than 45 years old. Logistic regression models were used to obtain the odds ratio (OR) and 95% confidence interval (CI). In the regression model, EOH frequency was included as a categorical variable (<5, 5–9, and ≥10 times per week) in the analysis, with the lowest level as the reference. Three models were employed in the analysis: Model 1 was unadjusted; Model 2 adjusted for age, BMI, and sex (except for the separate analyses in men and women); and Model 3 adjusted for age, BMI, sex (except for the separate analyses in men and women), smoking status, drinking status, physical activity, hypertension, dyslipidemia, and history of family diabetes. Trend tests were computed to study the relationship across each of the three levels of EOH frequency, entering the medians of each level as continuous variables in regression models.

A restricted cubic spline (RCS)^19^ was used to estimate the dose–response relationship between EOH frequency and the occurrence of T2DM, with the EOH frequency included as a continuous variable. Three knots (10th, 70th, and 100th percentiles) were selected in the cubic spline analysis, and 5 times/week was set as the reference. All analyses were conducted with SAS 9.4 (SAS Institute) and SPSS for Windows version 20.0 (IBM Corporation) software with a two‐sided test, which was reported as statistically significant at *p*_value_ ≤ 0.05.

## 3 RESULTS

### 3.1 Characteristics of the study population

Table 1 shows the characteristics of the study participants. A total of 13,904 participants (9230 males and 4574 females) were included in this analysis. The T2DM group had significantly higher average age, BMI, TC, TG, LDL‐C, SBP, DBP, FBG, OGTT 2 h blood glucose, and HbA1c levels compared to the non‐T2DM group. The T2DM group also had a higher proportion of individuals with hypertension, dyslipidemia, and a family history of T2DM than the non‐T2DM group. Finally, the T2DM group had a significantly lower average HDL‐C level than that of the non‐T2DM group, which was observed in both male and female subpopulations. A significantly higher proportion of T2DM participants in the total population were classified as smokers, which was also true in the male subpopulation. The T2DM group in the total population also had a significantly higher proportion of drinkers (42.8%).

**Table 1:** Characteristics of eligible participants in the analysis.

Characteristics	Total (*n* = 13,904)			Male (*n* = 9230)			Female (*n* = 4674)		
	Non‐T2DM	T2DM	*p*	Non‐T2DM	T2DM	*p*	Non‐T2DM	T2DM	*p*
Age, years	46.67 ± 9.69	51.13 ± 9.72	<0.01	46.22 ± 9.47	49.92 ± 9.58	<0.01	47.52 ± 10.04	54.5 ± 9.33	<0.01
Sex, *n* (%)			<0.01			‐			‐
Male	7864 (65.3)	1366 (73.5)		‐	‐		‐	‐	
Female	4182 (34.7)	492 (26.5)		‐	‐		‐	‐	
Smoking status, *n* (%)			<0.01			<0.01			0.571
Nonsmoker	7196 (59.8)	979 (52.7)		3134 (39.9)	497 (36.4)		4062 (97.2)	482 (98.0)	
Smoker/ex‐smoker	4843 (40.2)	879 (47.3)		4725 (60.1)	869 (63.6)		117 (2.8)	10 (2.0)	
Drinking status, *n* (%)			<0.01			0.823			<0.01
Nondrinker	7280 (60.5)	1063 (57.2)		3443 (43.8)	594 (43.5)		3836 (91.8)	469 (95.3)	
Drinker/ex‐drinker	4759 (39.5)	795 (42.8)		4416 (56.2)	772 (56.5)		343 (8.2)	23 (4.7)	
Physical activity, *n* (%)			0.328			0.572			0.127
Low	3973 (33.0)	646 (34.8)		3075 (39.1)	554 (40.6)		898 (21.5)	92 (18.7)	
Moderate	4037 (33.5)	607 (32.7)		2819 (35.9)	473 (34.6)		1217 (29.1)	134 (27.2)	
High	4024 (33.4)	605 (32.6)		1962 (25.0)	339 (24.8)		2062 (49.4)	266 (54.1)	
Eating out of home frequency/week			0.023			0.264			0.124
<5, *n* (%)	9007 (74.8)	1358 (73.1)		5218 (66.4)	903 (66.1)		3789 (90.7)	455 (92.5)	
5 − 9, *n* (%)	1776 (14.8)	267 (14.4)		1521 (19.4)	248 (18.2)		255 (6.1)	19 (3.9)	
≥10, *n* (%)	1255 (10.4)	233 (12.5)		1120 (14.3)	215 (15.7)		135 (3.2)	18 (3.7)	
Family history of T2DM, *n* (%)			<0.01			<0.01			<0.01
Yes	2553 (21.2)	518 (27.9)		1529 (19.5)	363 (26.6)		1024 (24.5)	155 (31.6)	
No	9471 (78.8)	1338 (72.1)		6321 (80.5)	1002 (73.4)		3149 (75.5)	336 (68.4)	
Hypertension, *n* (%)			<0.01			<0.01			<0.01
Yes	3728 (30.9)	1018 (54.8)		2771 (35.2)	771 (56.4)		3224 (77.1)	247 (50.2)	
No	8318 (69.1)	840 (45.2)		5093 (64.8)	595 (43.6)		957 (22.9)	245 (49.8)	
Dyslipidemia, *n* (%)			<0.01			<0.01			<0.01
Yes	5241 (43.5)	1202 (64.7)		4057 (51.6)	952 (69.7)		1184 (28.3)	250 (50.8)	
No	6805 (56.5)	656 (35.3)		3807 (48.4)	414 (30.3)		2997 (71.7)	242 (49.2)	
BMI, kg/m^2^	25.06 ± 3.46	26.69 ± 3.42	<0.01	25.67 ± 3.25	26.92 ± 3.28	<0.01	23.92 ± 3.56	26.07 ± 3.71	<0.01
TC, mmol/L	5.1 ± 0.98	5.5 ± 1.26	<0.01	5.14 ± 0.98	5.50 ± 1.31	<0.01	5.03 ± 0.99	5.51 ± 1.08	<0.01
TG, mmol/L	1.85 ± 1.65	2.87 ± 3.01	<0.01	2.11 ± 1.85	3.18 ± 3.35	<0.01	1.36 ± 1.03	2.00 ± 1.39	<0.01
HDL‐C, mmol/L	1.36 ± 0.35	1.28 ± 0.37	<0.01	1.28 ± 0.31	1.24 ± 0.38	<0.01	1.53 ± 0.36	1.41 ± 0.31	<0.01
LDL‐C, mmol/L	2.81 ± 0.76	2.93 ± 0.81	<0.01	2.85 ± 0.75	2.89 ± 0.81	0.017	2.75 ± 0.77	3.03 ± 0.81	<0.01
SBP, mmHg	125.88 ± 41.58	135.65 ± 18.12	<0.01	128.23 ± 49.51	135.90 ± 17.42	<0.01	121.46 ± 18.49	134.98 ± 19.92	<0.01
DBP, mmHg	78.61 ± 12.19	84.3 ± 12.55	<0.01	81.05 ± 11.90	86.01 ± 12.20	<0.01	74.02 ± 11.36	79.56 ± 12.30	<0.01
FBG, mmol/L	5.29 ± 0.55	7.82 ± 2.91	<0.01	5.33 ± 0.56	8.02 ± 3.02	<0.01	5.21 ± 0.53	7.26 ± 2.51	<0.01
OGTT 2 h blood glucose, mmol/L	6.81 ± 1.60	14.42 ± 5.13	<0.01	6.81 ± 1.63	14.61 ± 5.30	<0.01	6.80 ± 1.55	13.88 ± 4.60	<0.01
HbA1c, %	5.47 ± 0.37	6.88 ± 1.61	<0.01	5.48 ± 0.37	6.96 ± 1.64	<0.01	5.45 ± 0.38	6.67 ± 1.50	<0.01

*Note*: Continuous variables are presented as mean ± SD; categorical variables are shown as *n* (%). *p* Values were from Student's *t* test for continuous data and *χ* ^2^ test for categorical data.

Abbreviations: BMI, body mass index; DBP, diastolic blood pressure; FBG, fasting blood glucose HDL‐C, high‐density lipoprotein cholesterol; LDL‐C, low‐density lipoprotein cholesterol; OGTT, oral glucose tolerance test; SBP, systolic blood pressure; T2DM, type 2 diabetes mellitus; TC, total cholesterol; TG, triglyceride.

### 3.2 Association of EOH frequency and T2DM

Table 2 presents the association between the weekly frequency of EOH and T2DM. In the total population, participants with weekly EOH frequency ≥10 times showed a positive association with T2DM in the crude model (Model 1: OR = 1.23, 95% CI [1.06–1.43]), the model adjusted for age, BMI and sex (Model 2: OR = 1.36 95% CI [1.16–1.60]), and the fully adjusted model (Model 3: OR = 1.31, 95% CI [1.11–1.54]).

**Table 2:** Association between weekly frequency of eating out of home and type 2 diabetes mellitus.

EOH	Model 1			Model 2			Model 3		
	OR	95% CI	*p*	OR	95% CI	*p*	OR	95% CI	*p*
**Total (** ***n*** = **13,897)**									
<5 times (*n* = 10,366)	1 (Reference)			1 (Reference)			1 (Reference)		
5–9 times (*n* = 2043)	1.00	(0.87, 1.15)	0.968	1.10	(0.95, 1.28)	0.198	1.05	(0.90, 1.22)	0.564
≥10 times (*n* = 1488)	1.23	(1.06, 1.43)	0.007	1.36	(1.16, 1.60)	<0.01	1.31	(1.11, 1.54)	0.002
*P* trend			0.085			0.058			0.171
**Male (** ***n*** = **9225)**									
<5 times (*n* = 6121)	1 (Reference)			1 (Reference)			1 (Reference)		
5–9 times (*n* = 1,769)	0.94	(0.81, 1.10)	0.442	1.10	(0.94, 1.289)	0.244	1.05	(0.89, 1.23)	0.582
≥10 times (*n* = 1488)	1.11	(0.94, 1.31)	0.210	1.31	(1.11, 1.56)	0.002	1.26	(1.06, 1.51)	0.009
*P* trend			0.322			0.002			0.011
**Female (** ***n*** = **4672)**									
< 5 times (*n* = 4245)	1 (Reference)			1 (Reference)			1 (Reference)		
5–9 times (*n* = 274)	0.62	(0.39, 1.00)	0.049	0.96	(0.59, 1.57)	0.871	0.95	(0.57, 1.58)	0.845
≥ 10 times (*n* = 1488)	1.11	(0.67, 1.83)	0.682	0.67	(0.99, 2.81)	0.055	1.75	(1.03, 2.97)	0.039
*p* trend			0.097			0.097			0.075
**Age** ≤ **45 years (** ***n*** = **6300)**									
< 5 times (*n* = 4146)	1 (Reference)			1 (Reference)			1 (Reference)		
5–9 times (*n* = 1229)	1.50	(1.21, 1.86)	<0.01	1.21	(0.97, 1.52)	0.096	1.19	(0.94, 1.50)	0.147
≥ 10 times (*n* = 1488)	1.86	(1.49, 2.33)	<0.01	1.46	(1.15, 1.85)	0.002	1.41	(1.11, 1.80)	0.006
*p* trend			<0.01			0.002			0.005
**Age** > **45 years (** ***n*** = **7599)**									
0–5 times (*n* = 6220)	1 (Reference)			1 (Reference)			1 (Reference)		
5–9 times (*n* = 814)	0.98	(0.81, 1.20)	0.872	1.01	(0.82, 1.24)	0.958	0.93	(0.76, 1.15)	0.526
≥10 times (*n* = 1488)	1.24	(0.99, 1.54)	0.051	1.27	(1.01, 1.59)	0.040	1.22	(0.97, 1.54)	0.094
*p* trend			0.085			0.058			0.171

*Note*: Model 1: unadjusted. Model 2: adjusted for age, BMI, and sex (except for male and female population). Model 3: adjusted for age, BMI, sex (except for male and female population), smoking status, drinking status, physical activity, hypertension, dyslipidemia, and family history of type 2 diabetes mellitus. The odds ratio (OR) and 95% confidence interval (CI) were calculated using logistic regression models. A linear trend test was performed including the median value of each category of the categorical variable (<5, 5–9, and ≥10 times per week) as a continuous variable.

Abbreviations: EOH: eat out of home.

Positive associations between EOH ≥ 10 times/week and T2DM were also observed in participants ≤45 years old (Models 1, 2, and 3, *P*_trend_ < 0.05) and the male participants (Models 2 and 3, *p*_trend_ < 0.05). In the full‐adjusted model (Model 3), compared with the reference group, the OR of the group with EOH frequency ≥ 10 times/week was 1.41 (95% CI: [1.11–1.80], *p* < 0.01) in the participants ≤45 years old and 1.26 (95% CI: [1.06–1.51], *p* < 0.01) in the male participants.

In females, EOH ≥ 10 times/week was also correlated with increased odds of T2DM (Model 3: OR = 1.75, 95% CI: [1.03–2.97]), although the *p*_trend_ was >0.05. For individuals >45 years old, the EOH‐T2DM association was no longer significant based on the results of Model 3 (*P*_EOH≥10_ = 0.094 and *p*_trend_ = 0.171). Table S1 shows the association between the weekly frequency of EOH and the incidence of impaired fasting glucose (IFG)/impaired glucose tolerance (IGT).

### 3.3 Dose–response analysis of EOH frequency on the occurrence of T2DM

**Figure 1:**
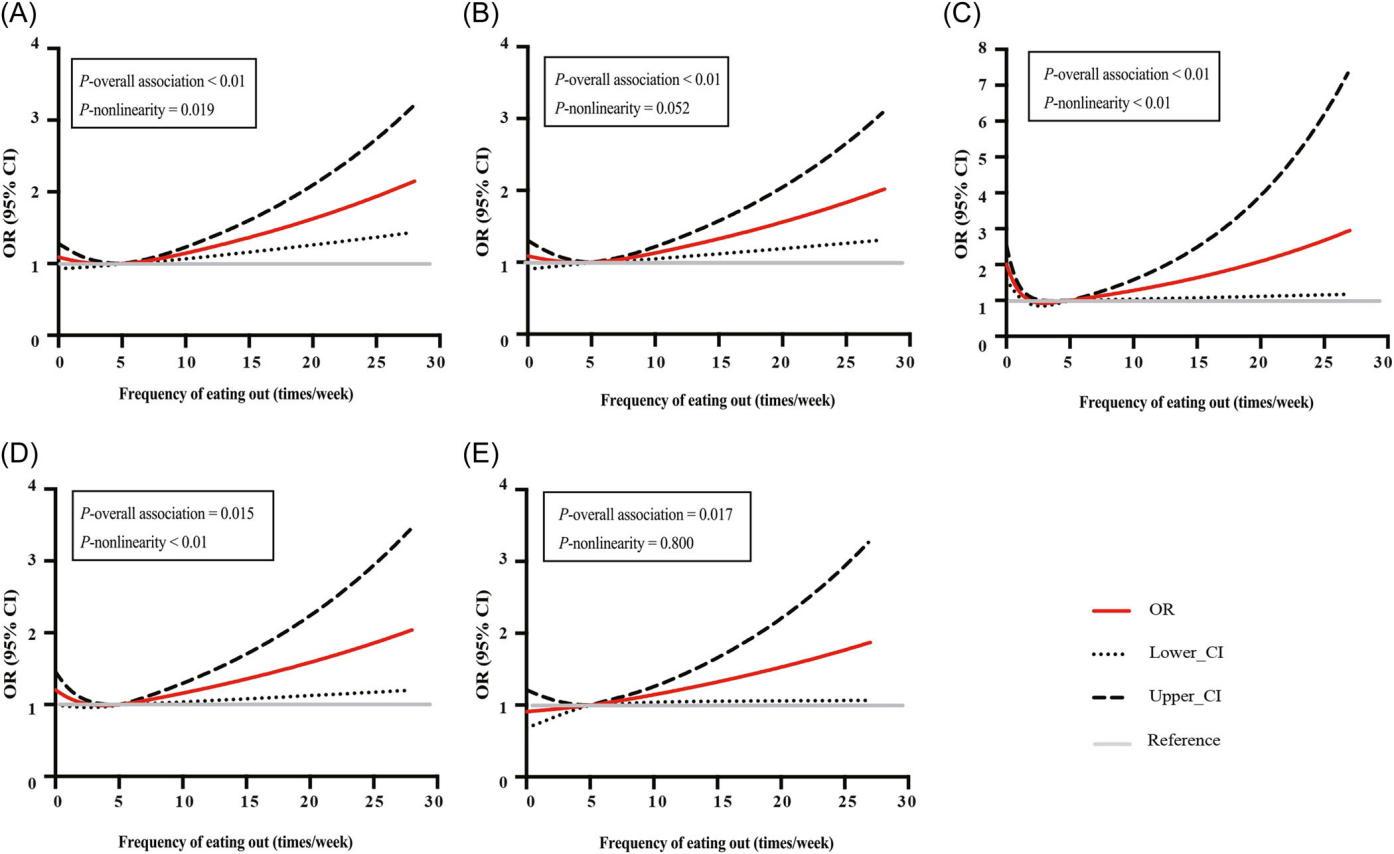
Dose–response associations between weekly frequency of eating out of home (EOH) and type 2 diabetes mellitus (T2DM) in (A) the total population, (B) male population, (C) female population, (D) population > 45 years old, and (E) population ≤45 years old. The fully adjusted model included age, gender (except for male and female population), body mass index (BMI), smoking status, drinking status, physical activity, hypertension, dyslipidemia, and family history of T2DM. CI, confidence interval; OR, odds ratio.

Figure 1 shows the dose–response relationship between EOH frequency and the OR of T2DM. The RCS curves reveal significant J‐shaped EOH frequency‐T2DM associations in the total population, male participants, female participants, and participants >45 years old (Figure 1A–D, *p*_overall association_ < 0.05 and *p*_nonlinearity_ ≤0.05). According to the curves, 5 or fewer times per week of EOH did not increase the odds of T2DM, while when EOH was more than 5 times per week, the OR of T2DM significantly increased in a dose‐related manner (Figure 1A‐D). In addition, a positive and linear EOH‐T2DM association could be observed in the participants ≤45 years old (Figure 1E, *p*_overall association_ = 0.017 and *p*_nonlinearity_ >0.05), without a threshold.

## 4 DISCUSSION

Using data from 13,904 Chinese urban workers collected from 11 health centers located in different big cities of China, the current study analyzed the association between EOH frequency and the occurrence of T2DM. A positive EOH‐T2DM association was identified in the total population, individuals ≤ 45 years old, and in both the male and female urban worker subpopulations (Table 2). An EOH frequency of 5 times/week seems to be a threshold for a significant increase in the odds of T2DM. Higher EOH frequencies were associated with increased odds of T2DM in a dose‐related manner in the total population and in almost all subgroups, except for a significant positive linear association in urban workers ≤45 years old (Figure 1E).

The relationship between EOH and the incidence of T2DM has been previously reported in both Western countries^14^ and in China.^6^ However, the evidence is quite limited. In particular, for Chinese urban workers facing significant working pressure, lifestyle changes, and higher frequency of EOH, no previous study has specifically explored the EOH‐T2DM association. In our analysis, EOH was generally positively associated with T2DM, which is consistent with previous studies.^6^ The effect of EOH on T2DM was independent of sex in our results (Table 2), which is a finding that differs from the findings in a previous study of rural Chinese workers.^15^ We also found that age might have a moderating effect on the EOH‐T2DM association, i.e., the association was more robust in people ≤45 years old than it was in those > 45 years old (Table 2). Age was previously identified to be inversely associated with EOH frequency,^22^ which may be a possible explanation for the age‐group differences found in this study. This result suggests that unhealthful diets should be a concern of increased risk of diabetes in young people. Adults ≤45 years old constitute the largest portion of China's labor force. We speculate that EOH of 5 times/week may be associated with eating takeout or cafeteria food once a day during a 5‐day work week. The data in this study indicate that limiting EOF frequency to no more than 5 times/week was not accompanied by an increased T2DM incidence in the total population and almost all subgroups (Figure 1). Therefore, providing a healthful lunch at the workplace would help to ensure that the T2DM risk was minimized.

This recommendation stems from the likelihood that the increased risk of T2DM is associated with suboptimal food consumption during EOH events and a resulting nutritional imbalance. First, compared with meals at home, foods consumed out of home tend to be high in caloric value but low in micronutrients, such as minerals and vitamins (e.g., B group vitamins, folate, vitamin C, and vitamin D).^8^, ^23^ Excessive caloric intake is associated with overweight, obesity, T2DM, and other metabolic diseases.^24^ Adequate vitamin and mineral intake may improve cardiometabolic health.^25^ Specifically, excessive caloric intake accompanied by inadequate micronutrient intake of EOH has been shown to increase the BMI ^26^ and cardiovascular disease risk.^27^

Second, according to the National Diet and Nutrition Survey of the UK, EOH was associated with higher ultra‐processed food consumption.^28^ Ultra‐processed foods are high in saturated fat, sugar, and additives. The consumption of ultra‐processed foods increases the risk of T2DM in a dose‐related manner.^29^ Moreover, EOH may have food safety issues. Compared with meals prepared at home, foods from unknown sources may have an increased risk of contamination with microbiological hazards, which are also a risk factor for T2DM.^30^

Third, EOH may increase the intake of beverages and sodium. Individuals who have meals out of home tend to consume more beverages such as alcohol, beer, and sugar‐sweetened beverages.^9^ Although the influence of alcohol consumption on T2DM incidence is still inconclusive,^31^ consumption of sugar‐sweetened beverages is positively associated with risks of obesity and T2DM.^32^ Sugar‐sweetened beverage consumption was also found to increase dietary sodium intake in a cross‐sectional analysis of Chinese children and adolescents.^33^ Excessive sodium intake is thought to activate signaling pathways involved in obesity‐related insulin resistance.^34^ In a meta‐analysis of studies from 25 countries, T2DM individuals were shown to have higher sodium intake than non‐T2DM individuals.^35^

It should be noted that EOH may actually increase the diversity of food intake, especially an increased intake of aquatic products (e.g., fish and seafood), which is generally regarded to be beneficial for improving cardiometabolic health.^36^ A survey performed among Chinese young persons showed that although EOH reduced the intake of vegetables and fruits, EOH was positively associated with an increase in the weekly intake of meat and aquatic products.^37^ Therefore, from this perspective, if EOH meals result in increased food diversity while reducing harmful ingredients such as oil, salt, and sugar, it could be a net benefit for such eaters.

This study is the first attempt to assess the association between EOH frequency and T2DM in a population of Chinese urban workers using data from multiple centers in nine major cities of China. Our study indicates the importance of providing healthful food during the working day as a way to help manage the risk of diabetes.

This study has several limitations. First, our study has no record of the time of eating out. For example, compared with rural workers, more urban workers tend to eat breakfast outside of home.^15^ Breakfast quality and frequency have a direct relation with appetite control and blood glucose control.^38^ Findings from the China Health and Nutrition Survey indicated that overeating at dinner had more influence on T2DM incidence than overeating at other meals.^39^ In our analysis, it is unknown which meal out of home was associated with a higher odds ratio of T2DM.

Second, our study has no record of the daily types of foods or snacks participants ate, nor did it assess participants' daily calorie intake, which is also one of the factors that influence the incidence of T2DM.

Third, the participants involved in the current study are mainly middle‐aged workers, and conclusions drawn from the current analysis cannot be directly generalized to older people.

Fourth, our findings cannot be simply generalized to infer the effects on persons living in urban areas, especially considering that the rapid nutrition transition taking place during recent decades in China may not have been homogeneous across the country.

Fifth, the cross‐sectional design of the current study cannot determine the causal relationship between EOH and T2D.

Finally, although the participants in this study have similar socioeconomic status (i.e., income, and education), the influence of their occupation on the association of diabetes risk was not fully addressed.

In conclusion, our study provided evidence that a high frequency of EOH was associated with increased odds of T2DM in Chinese urban workers. The positive association between EOH and T2DM was independent of sex, and the association was stronger in individuals ≤45 years of age. EOH frequency of about 5 times/week seemed to be a threshold for the increased odds of T2DM. Well‐designed cohort studies are needed to validate the findings of this observational study.

Our study suggests that more nutrition education and promotion are needed to improve the eating behavior and diet quality (e.g., diet composition and diversity) of Chinese urban workers.

## AUTHOR CONTRIBUTIONS

All authors contributed to the study's conception and design. Ping Zeng and Jingjing He conceived and designed the study. Ping Zeng, Fangyan Chen, Sitong Wan, Shuyan Wang, Jinjuan Hao, Ke Sun, Annan Liu, and Ling Zhu were all involved in the data collection. Fangyan Chen, Sitong Wan, Shuyan Wang, and Jingjing He compiled the data before analysis. Sitong Wan and Jingjing He conducted data analysis. Fangyan Chen, Sitong Wan, Shuyan Wang, and Jingjing He drafted the manuscript. Ping Zeng, Fangyan Chen, Annan Liu, and Ke Sun revised the manuscript. All authors read and approved the final manuscript.

## CONFLICT OF INTEREST STATEMENT

The authors declare no conflict of interest.

## ETHICS STATEMENT

The study was approved by the Medical Ethics Committee of the Beijing Hospital (approval No.: BJYYEC‐KY104‐01).

## Supporting information

Supporting information.

## Data Availability

Original data are available upon reasonable request. To request access to the data, contact Ping Zeng.
