# Supplementary material for: Association of eating out of home and type 2 diabetes mellitus in Chinese urban workers: A nationwide study
Source: Chronic Dis Transl Med. 2024 Jun 13;11(1):69–77. doi: 10.1002/cdt3.136 (PMC11880116; doi:10.1002/cdt3.136)
Supplement: Supplementary file 1 — Supporting information. [file CDT3-11-69-s001.docx]

**Supplementary Table S1.** Multivariate-adjusted OR and 95% CI for incidence of IGT/IFG according to the weekly frequency of eating out.

| Items | Model 1 | | |  | Model 2 | | |  | Model 3 | | |
| --- | --- | --- | --- | --- | --- | --- | --- | --- | --- | --- | --- |
|  | OR | 95% CI | *P* |  | OR | 95% CI | *P* |  | OR | 95% CI | *P* |
|  |  |  |  |  |  |  |  |  |  |  |  |
| **Total (*n* = 13,897)** |  |  |  |  |  |  |  |  |  |  |  |
| <5 times (*n* = 10,366) | 1 (Reference) | | |  | 1 (Reference) | | |  | 1 (Reference) | | |
| 5-9 times (*n* = 2,043) | 0.88 | (0.72,1.07) | 0.199 |  | 1.08 | (0.87,1.33) | 0.198 |  | 1.05 | (0.90,1.22) | 0.564 |
| ≥ 10 times (*n* = 1,488) | 0.86 | (0.68,1.09) | 0.203 |  | 1.07 | (0.83,1.37) | <0.01 |  | 1.31 | (1.11,1.54) | 0.002 |
| *P* trend |  |  | 0.120 |  |  |  | 0.058 |  |  |  | 0.171 |
|  |  |  |  |  |  |  |  |  |  |  |  |
| **Age ≤ 45 (*n* = 6,300)** |  |  |  |  |  |  |  |  |  |  |  |
| < 5 times (*n* = 4,146) | 1 (Reference) | | |  | 1 (Reference) | | |  | 1 (Reference) | | |
| 5-9 times (*n* = 1,229) | 1.50 | (1.09,2.06) | 0.014 |  | 1.26 | (0.90,1.75) | 0.096 |  | 1.31 | (0.93,1.83) | 0.147 |
| ≥ 10 times (*n* = 923) | 1.35 | (0.93,2.00) | 0.115 |  | 1.09 | (0.74,1.61) | 0.002 |  | 1.09 | (0.73,1.61) | 0.006 |
| *P* trend |  |  | 0.046 |  |  |  | 0.002 |  |  |  | 0.005 |
|  |  |  |  |  |  |  |  |  |  |  |  |
| **Age > 45 (*n* = 7,599)** |  |  |  |  |  |  |  |  |  |  |  |
| 0-5 times (*n* = 6,220) | 1 (Reference) | | |  | 1 (Reference) | | |  | 1 (Reference) | | |
| 5-9 times (*n* = 814) | 0.76 | (0.49,1.16) | 0.199 |  | 1.01 | (0.79,1.28) | 0.958 |  | 0.98 | (0.76,1.25) | 0.526 |
| ≥ 10 times (*n* = 565) | 0.82 | (0.51,1.32) | 0.424 |  | 1.01 | (0.76,1.34) | 0.040 |  | 0.97 | (0.73,1.30) | 0.094 |
| *P* trend |  |  | 0.258 |  |  |  | 0.058 |  |  |  | 0.171 |
|  |  |  |  |  |  |  |  |  |  |  |  |

Model 1: unadjusted.
Model 2: adjusted for age, BMI and sex (except for male and female population).

Model 3: adjusted for age, BMI, sex (except for male and female population), smoking status, drinking status, physical activity, hypertension, dyslipidemia, and family history of type 2 diabetes mellitus.

Odds ratio (OR) and 95% confidence interval (CI) were calculated using logistic regression models. Linear trend test was performed including the median value of each category of the categorical variable (<5, 5-9 and ≥10 times per week) as a continuous variable.
